# Supplementary material for: Pharmacological Activation of Potassium Channel Kv11.1 with NS1643 Attenuates Triple Negative Breast Cancer Cell Migration by Promoting the Dephosphorylation of Caveolin-1
Source: Cells. 2022 Aug 8;11(15):2461. doi: 10.3390/cells11152461 (PMC9368491; doi:10.3390/cells11152461)
Supplement: Supplementary file 1 [file cells-11-02461-s001.zip › cells-1839545-supplementary.pdf]

Figure S1

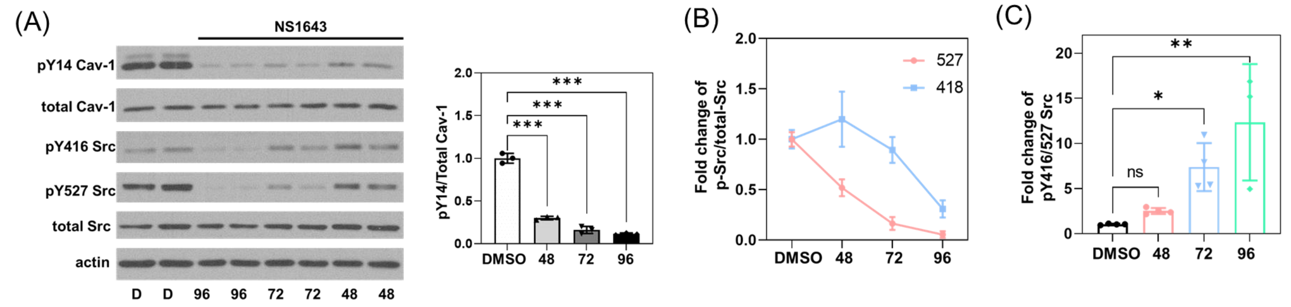

Figure S1. Dephosphorylation of Cav-1 induced by NS1643 is not dependent on inhibition of Src activity.

(A) MDA-MB-231 cells treated with DMSO or 50 μM NS1643 for 48, 72 and 96 h were collected, lysed and subjected to western blotting. The dephosphorylation of Cav-1 induced by NS1643 increased with time.

(B) The phosphorylation of both Y416 and Y527 in Src decreased with NS1643.

(C) The activity of Src kinase represented by the ratio of phosphorylation of 416 and 527 sites increased with NS1643 treatment which suggests that the dephosphorylation of Cav-1 is not due to the inhibition of Src kinase activity. Data are mean ± SD, n=4; ns=not significant, *p<0.05, **p<0.005, ***p<0.001 by ANOVA.

Table S1

Label	126	127N	128C	129N	130C	131
Sample	DMSO1	DMSO2	DMSO3	NS1	NS2	NS3

Table S1. Scheme for the sample TMT 6 labeling.

MDA-MB-231 cells treated with DMSO or 50μM NS1643 for 48 h were lysed and immunoprecipitated with anti-β-catenin antibody covalently conjugated with magnetic beads. The samples were eluted and grouped into six batches and labeled with TMT6plex Isobaric Label Reagent Set according to the manufacturer's instructions.

Video S1. Single cell tracking of cells expressing Cav-1WT-CFP.
MCF-7 cells transfected with CFP tagged Cav-1WT were treated with DMSO or 50 μM NS1643 and imaged with Olympus VivaView Fluorescence Microscope every 6 min to track the movement of individual cells for 4 h. Image sequences were processed by ImageJ to form single cell tracking video. The motility of cells expressing Cav-1WT-CFP was inhibited by NS1643.

Video S2. Single cell tracking of cells expressing Cav-1Y14D-CFP.
MCF-7 cells transfected with CFP tagged Cav-1Y14D mutant were treated with DMSO or 50 μM NS1643 and imaged every 6 min to track the movement of individual cells for 4 h. Image sequences were processed by ImageJ to form single cell tracking video. The motility of cells expressing Cav-1Y14D-CFP was not affected by NS1643.

Video S3. Single cell tracking of cells expressing Cav-1Y14F-CFP.
MCF-7 cells transfected with CFP tagged Cav-1Y14F mutant were treated with DMSO or 50 μM NS1643 and imaged every 6 min to track the movement of individual cells for 4 h. Image sequences were processed by ImageJ to form single cell tracking video. The motility of cells expressing Cav-1Y14F-CFP was not affected by NS1643.
